# Wound Bed Temperature has Potential to Indicate Infection Status: A Cross‐Sectional Study

**DOI:** 10.1111/wrr.70072

**Published:** 2025-08-01

**Authors:** Adam R. Collins, Gerard M. O'Connor, Darragh A. Ryan, Molly Parmeter, Sean Dinneen, Georgina Gethin

**Affiliations:** ^1^ Physics, School of Natural Sciences University of Galway Galway Ireland; ^2^ Cúram Research Ireland Centre for Medical Devices University of Galway Galway Ireland; ^3^ Alliance for Research and Innovation in Wounds University of Galway Galway Ireland; ^4^ School of Medicine University of Galway Galway Ireland; ^5^ Centre for Diabetes, Endocrinology and Metabolism Galway University Hospitals Galway Ireland; ^6^ School of Nursing and Midwifery University of Galway Galway Ireland

**Keywords:** inflammation, thermal imaging, wound assessment, wound infection diagnosis, wound temperature

## Abstract

This study investigates the potential of wound bed temperature, measured using an IR camera, to aid in the clinical assessment of chronic wounds. The study captured thermal images from 267 patients with chronic wounds (diabetic foot ulcers, pressure ulcers, venous leg ulcers and arterial ulcers) with corresponding photographic images and clinical data. Temperature measurements were extracted from thermal images, focusing on both the centre of the wound and the surrounding periwound skin. Statistical analyses were employed to evaluate the relationship between wound temperature distribution and clinical diagnosis. The results showed a strong correlation between wound centre temperature and the average temperature across the entire wound (*R* ^2^ = 0.977). This indicates that a single‐point measurement is representative of the entire wound, simplifying wound temperature assessment. A fair correlation was found between the temperature difference between the wound and periwound and the clinician's assessment of infection status (Pearson coefficient = 0.32). The study concludes that thermal imaging holds promise as a supplementary tool for clinicians in assessing chronic wound status, especially in cases where infection is unclear. It is a low‐cost, non‐contact, and easy‐to‐use technique.

**Abbreviations**
AD: Alzheimer's disease
AU: arterial ulcer
CHF: chronic heart failure
CI: confidence interval
CKD: chronic kidney disease
COPD: chronic obstructive pulmonary disease
DFU: diabetic foot ulcer
HChol: hypercholesterolaemia
HTN: hypertension
MAVLUs: mixed arterio‐venous leg ulcers
OTH: other ulcer
PAD: peripheral arterial disease
PU: pressure ulcer
T1DM: type 1 diabetes
T2DM: type 2 diabetes
VLU: venous leg ulcer

## 1 Introduction

As people live longer, the prevalence of chronic diseases such as diabetes, cardiovascular disease, hypertension, and respiratory diseases is projected to increase [1]. Analogous to this, the prevalence of chronic wounds such as diabetic foot ulcers (DFUs), pressure ulcers (PUs), and venous leg ulcers (VLUs) is also projected to increase [2]. Chronic disease and chronic wounds pose a significant humanistic and economic burden to individuals and society as a whole [3]. It is imperative, therefore, to research effective, efficient, and equitable ways to prevent and treat wounds to the benefit of all.

Appropriate wound management starts with a comprehensive assessment of the wound and of patient co‐factors. However, a major challenge remains in that most wound bed assessment remains reliant on subjective visual inspection by the attending clinician. This can be supported by some objective measurements, such as wound size, but ultimately the quality and interpretation of the assessment is reliant on the knowledge and expertise of the clinician. It is recognised that devices are currently being developed to aid this assessment process, for example by identifying the presence of bacteria within the wound, but they can be expensive and randomised controlled trials are required to determine the benefits of such technologies on wound and patient outcomes. Therefore, there is a need to continue to explore inexpensive and widely accessible alternatives. Monitoring of wound temperature may hold promise in achieving this.

Temperature is a critical parameter in healthcare for understanding illness and monitoring treatment responses. A core body temperature about 1°C above the normal baseline of 37°C is a reliable indicator of fever [4]. Additionally, localised inflammatory responses often result in increased blood flow, causing a rise in temperature at the affected area. Skin temperature is generally lower than core body temperature and is more variable and more sensitive to environmental conditions. Previous studies have shown that wounds exhibiting elevated temperatures in the wound area and the periwound skin are associated with infections and poor healing outcomes [5, 6]. A minimum temperature increase of 1.1°C has been identified as a threshold for concern [6]. A low temperature reading from a wound is also a sign of poor healing. Ischaemic wounds will exhibit a lower temperature than non‐ischaemic wounds due to restricted blood flow [7].

Quantitative thermal imaging has been used in medicine for over 30 years [8]. Over this period, thermal imagers have undergone significant advancements, transitioning from bulky, actively cooled devices with 10 or fewer sensors to compact, uncooled systems boasting thousands of sensors. This miniaturisation has enabled integration into a smartphone for unrestricted use. Consequently, the devices have reached a form factor and price point enabling their use in day‐to‐day care of illness. Standards for medical thermal imaging are under development but are not widely used. Of note is the Glamorgan Protocol, which sought to standardise the capture of thermal images of the human body, reduce measurement error, and develop a database of thermal images of healthy examples of the human body [9, 10].

### 1.1 How IR Temperature Measurement Works

Understanding an object's emitted infrared (IR) radiation is necessary to interpret thermal images and recognise their strengths and limitations. All objects emit IR radiation. The amount of radiation emitted depends on the temperature of the body and how well the surface emits IR radiation, known as the emissivity of the surface. Thus, if the emissivity is known, the temperature of the body can be determined by measuring the amount of emitted IR radiation. A thermal camera measures IR radiation emitted from an object by using focusing optics to capture the emitted radiation and focus it onto an IR sensor, where it is converted to an electrical signal. Converting this signal to a temperature value relies on a user‐defined emissivity value for the target (between 0 and 1). To improve accuracy, some thermal cameras also account for additional variables, such as room temperature and humidity, the camera's own temperature, and the distance from the target. These factors help correct for environmental influences and enhance the reliability of the measurements.

In an ideal case, the signal measured (*U*) by the IR sensor, from which the thermal image is produced, is a function of the object temperature (*T*_obj_) and the emissivity (*ε*) of the surface, given by the Stefan‐Boltzmann equation:

$$U\sim \epsilon T_{\mathrm{obj}}^{4}$$

Practically, the signal measured by the IR detector is a function of IR energy from the surroundings reflecting off the target and emission from the device optics. A term to account for IR reflections from the target (*T*_amb_) and self‐radiation of the optics in the IR camera (*T*_optics_) is required. Here, *R* is the reflectivity of the target at the wavelengths of interest:

$$U\sim \epsilon {T_{\mathrm{obj}}}^{4}+R{T_{\mathrm{amb}}}^{4}+{\epsilon_{\text{optics}}T_{\text{optics}}}^{4}$$

Knowledge of *T*_amb_ and *T*_optics_ are needed to make an absolute measurement of wound temperature. The transmission of IR wavelengths through the atmosphere is dependent on the air temperature and humidity; hence, the temperature and humidity of the room are required, along with the distance from the target to the camera. IR radiation is not emitted equally in every direction; hence, the measured signal and apparent temperature will reduce as the viewing angle (θ) increases [11]. To avoid this effect, images should be captured perpendicular to the target surface.

Incandescent lighting and sunlight emit appreciable amounts of energy at wavelengths 8–14 μm, which is the region in which the thermal camera is sensitive. Such emissions can reflect from the target into the camera, causing the target temperature to appear higher.

Finally, the emissivity of human skin will vary with each patient. Clean skin is assumed to have an emissivity of 0.98 [12]. The emissivity of unclean skin, or dry flaky skin, will be reduced. The emissivity of wounds and intact skin is not equal. A study of the emissivity of wounds found wounds have an emissivity 0.01–0.03 higher than intact skin [12]. This introduces an error of approximately 0.05°C if not corrected for when taking measurements from thermal images.

**FIGURE 1:**
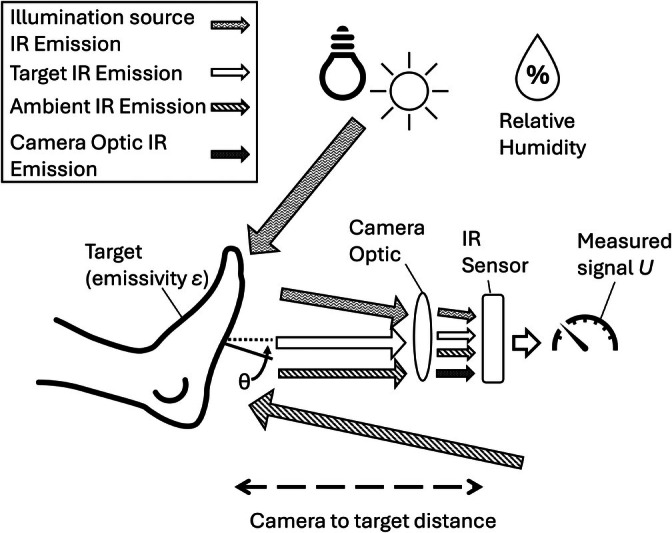
Elements contributing to the measured signal U when capturing a thermal IR image.

Figure 1 summarises the sources of error in IR thermal imaging. Ambient temperature and humidity can be measured; however, each of these measurements introduces some level of uncertainty, which contributes to the overall uncertainty in the measured signal *U*. Allowing time for the camera to acclimatise to the room temperature allows the user to assume the camera temperature and room temperature are equal, but some uncertainty in the precise temperature of the camera optic remains. Efforts can be made to minimise the viewing angle, θ, but for infirm patients, lifting a limb may pose challenges. Absolute skin temperature measurements from typical commercial thermal cameras can have uncertainties as high as ±2°C [13] depending on environmental conditions and image capture configuration. Consequently, accurately identifying localised wound inflammation, which typically manifests as temperature changes of approximately 2°C, is difficult due to this inherent measurement uncertainty. With a high‐end camera and an optimised and tightly controlled imaging setup, the uncertainty can be reduced to ±0.5°C [14]. This improvement is achieved through the use of reference radiators captured within the thermal image alongside the target [15]. A reference radiator is a calibration target that is set to a known temperature, and the surface of the target has a known emissivity. This allows the user to check the camera calibration and environmental conditions in real‐time or during data extraction. Such a controlled imaging setup would be difficult to implement in a wound clinic on infirm patients.

The accuracy limitations of thermal imaging can be mitigated by making relative temperature measurements of different regions within the same image, rather than relying on absolute temperature readings. This approach reduces the impact of environmental conditions as they can be assumed to be equal within the same image, particularly when examining adjacent regions on the same object. For wound monitoring, this allows different regions of the wound and the surrounding skin to be compared and used to determine wound condition. This is the approach used in this study.

While we may be able to advance the use of thermal images and temperature monitoring in wound assessment, it is critical to know the value of this to the attending clinician in the decision‐making process. In the future, the benefits or not of such information should be determined based on patient outcomes. This current study proposed to expand on previous work and determine to what extent thermal images correlated with clinician diagnosis of wound status and use this information in our quest to know if wound bed temperature measurement could provide a realistic aid to the clinician in assessing wound status.

## 2 Objectives

1. Establish a database of wound temperature distributions and corresponding wound status, derived from simultaneously acquired thermal images and wound photographs.
2. Determine the correlation between a single‐point temperature measurement at the wound's centre and the mean temperature of the entire wound area.
3. Evaluate the relationship between the temperature differential between the wound's centre and the surrounding periwound tissue, and the clinically assessed infection or healing status.

## 3 Material and Methods

### 3.1 Study Design

A prospective, observational study design was followed.

### 3.2 Eligibility Criteria

Adult patients with current, open chronic wounds such as VLUs, DFUs, PUs, or MAVLUs were eligible for inclusion. Additionally, patients must have been able to understand the nature and purpose of the study and provide informed consent.

This study complies with the ethical rules for human experimentation that are stated in the 1975 Declaration of Helsinki. Ethical approval was granted by the local Research Ethics committee of the Health Service Executive, reference number C.A. 2643. A site research assistant informed the patient of the study, provided an information leaflet, and gained informed consent.

Clinicians in both sites were experienced in wound management and had worked in both settings for a significant length of time, so were considered experts in the area. Data collection took place during regular podiatry and vascular clinics in June, July, and August across three consecutive years (2021, 2022, and 2023). Wound infection diagnoses were based on clinician judgement, considering wound bed characteristics and patient factors, in line with standard practice.

A set of patient data was recorded including: age, gender, diabetes status, site of wound, wound duration, wound infection status, aetiology, ulcer stage, wound healing status (healing, indeterminate or not healing), current dressing regime, underlying conditions, and whether debridement was performed or not.

### 3.3 Sample Size

As an observational study, we did not set a sample size but were guided by the potential number of patients attending the respective clinics and the need to build a database that would help answer the research question. After 3 months of data collection each year, we noted a significant amount of repeat patients with few 'new' patients presenting, and thus at this point, data collection ceased.

### 3.4 Thermal Imaging Methodology

The methodology was designed to capture consistent and high‐quality images, while minimising any patient discomfort and disruption to the wound clinic.

Eligible patients had a visual and thermal image of their wound recorded using a FLIR one pro camera (Flir Inc., Wilsonville, OR, USA) smartphone attachment. The camera simultaneously captures both images. The resolution of the thermal sensor is 160 × 120 pixels. The thermal camera has a minimum working distance of 150 mm. Images were captured at this distance where possible to maximise resolution in the wound in the thermal image.

The camera was allowed at least a 3‐min warm‐up period once it was powered on to reach stabilisation. The temperature and humidity of the room were measured using a digital thermometer and hygrometer. The viewing angle was kept as low as feasible considering the mobility of the patient. Care was taken to ensure no sunlight or incandescent lightbulbs were illuminating the target when the image was captured.

The images were recorded after the patient had their wound dressing removed and excess exudate cleaned. In most cases, images were taken after wound debridement was conducted. Excess dead skin on the wound surface can result in erroneously cool temperature readings as dead skin has a lower emissivity than living skin [12]. Images were recorded without delay to minimise any convective cooling of the wound surface.

The images were anonymised, uploaded to cloud storage, and then deleted from the device. A round 1 cm diameter sticker was placed next to the wound to act as a scale bar for the captured images.

### 3.5 Data Extraction Methodology

The temperature was extracted from the thermal images using FLIR thermal studio software. The values for room temperature, humidity, and distance to the target were entered into the software for each image. For measurements on intact skin, an emissivity value of 0.98 was used; for wounded skin, a value of 0.982 was used. The researcher extracting temperature measurements was blinded to the clinical infection status, mitigating potential bias in data analysis. Spot measurements were taken from the centre of the wound using the spot measurement tool. The mean temperature was measured using the circular fit tool. The circular measurement tool was adjusted to the wound for the best fit. The edge of the wound was defined by the epithelial tissue. Wound area was calculated by multiplying the largest horizontal measurement by the largest vertical measurement measured from the visual image. The scale sticker (1 cm diameter) in the visual image was used to determine scale.

**FIGURE 2:**
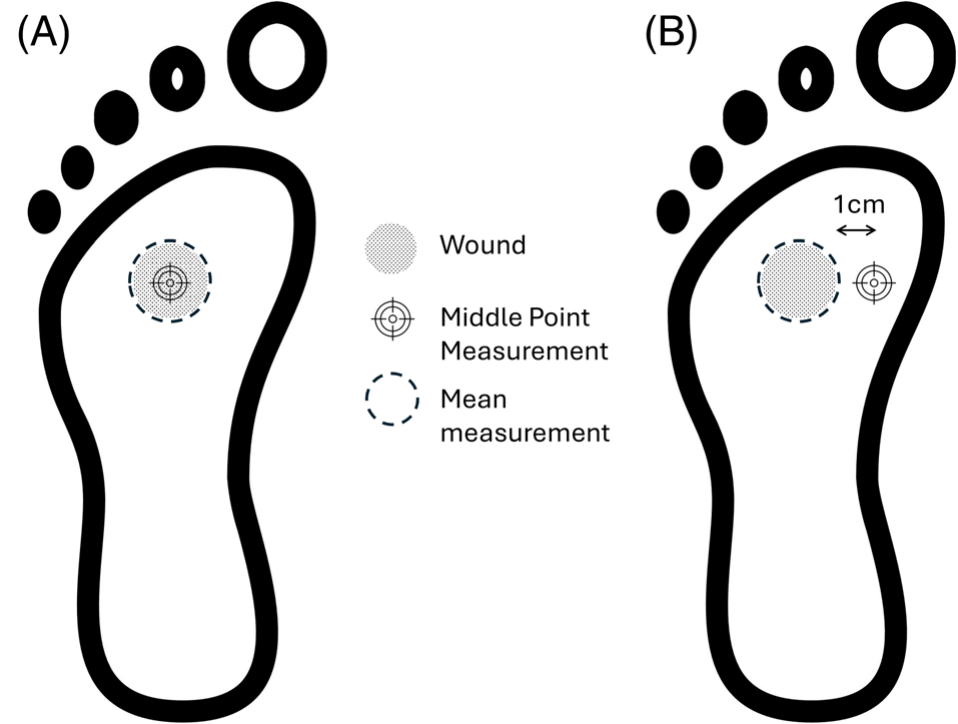
Temperature measurement methodology for thermal images. (a) shows the how measurements were made for comparison between wound mean temperature and wound middle temperature. (b) shows how the measurements were made for determining ΔT between the wound and periwound.

When measuring the ΔT value between the wound temperature and the periwound, the average wound temperature was measured using the circular measurement tool. The spot measurement tool was used to measure the healthy skin temperature 1 cm from the edge of the wound. Limb temperature naturally decreases as you move away from the trunk of the body; hence, the healthy skin measurement was made laterally along the foot. Figure 2 demonstrates the data measurement methodology. The mean temperature of the wound (T_mean_) was subtracted from (T_1cm_) and the absolute value of this difference ΔT_|(T1cm‐Tmean)|_ was calculated and used to determine the relationship with wound infection status.

The measurement methodology of a single lateral periwound temperature measurement was chosen for consistency and clinical practicality. While an average from multiple periwound points might offer a more comprehensive thermal profile and potentially alter the ΔT value, this approach is suited for routine use.

### 3.6 Data Analysis

Two quantitative statistical tools were used, the coefficient of determination and Bland–Altman analysis, to quantify the relationship between the middle temperature of the wound and the mean temperature.

The coefficient of determination (*R* ^2^) is used to calculate the proportion of variation in a dependent variable (e.g., middle temperature) which can be explained by an independent variable (e.g., mean temperature). The *R* ^2^ value can be determined from a plot of the dependent variable against the independent variable. The *R* ^2^ ranges from 0 to 1, with a higher value suggesting a stronger explanatory relationship between the two variables.

Correlation quantifies the degree to which two variables are related. But a high correlation does not necessarily indicate good agreement between the two methods. Bland–Altman analysis quantifies the agreement between two quantitative measurements. The mean of the two quantitative measurements is plotted on the *x* axis (Bland–Altman Mean) and the difference is plotted on the *y* axis.

For quantifying the correlation between infection status and ΔT_|(T1cm‐Tmean)|_ the Pearson coefficient was used. The Pearson coefficient value can be interpreted according to Table 1 [16].

**TABLE 1:** Interpretation of Pearson coefficient.

Pearson coefficient value	Strength of correlation
< 0.25	Little to no relationship
0.25–0.50	Fair relationship
0.50–0.75	Moderate to good relationship
> 0.75	Good to excellent relationship

The *R* ^2^ and Bland–Altman analysis were chosen because they allow us to not only assess how well the middle temperature explains the variation in mean temperature but also to determine if the two methods provide consistent results. The Pearson coefficient was selected for its ability to assess linear relationships in the context of infection status.

## 4 Results

### 4.1 Patient and Wound Characteristics

272 images were captured. Three images did not have associated patient data and were excluded. Two images contained wounds which were not easily visible and were excluded. One image contained multiple wounds which were treated as separate entries. The final dataset contained 268 wounds from 267 patients [17].

**FIGURE 3:**
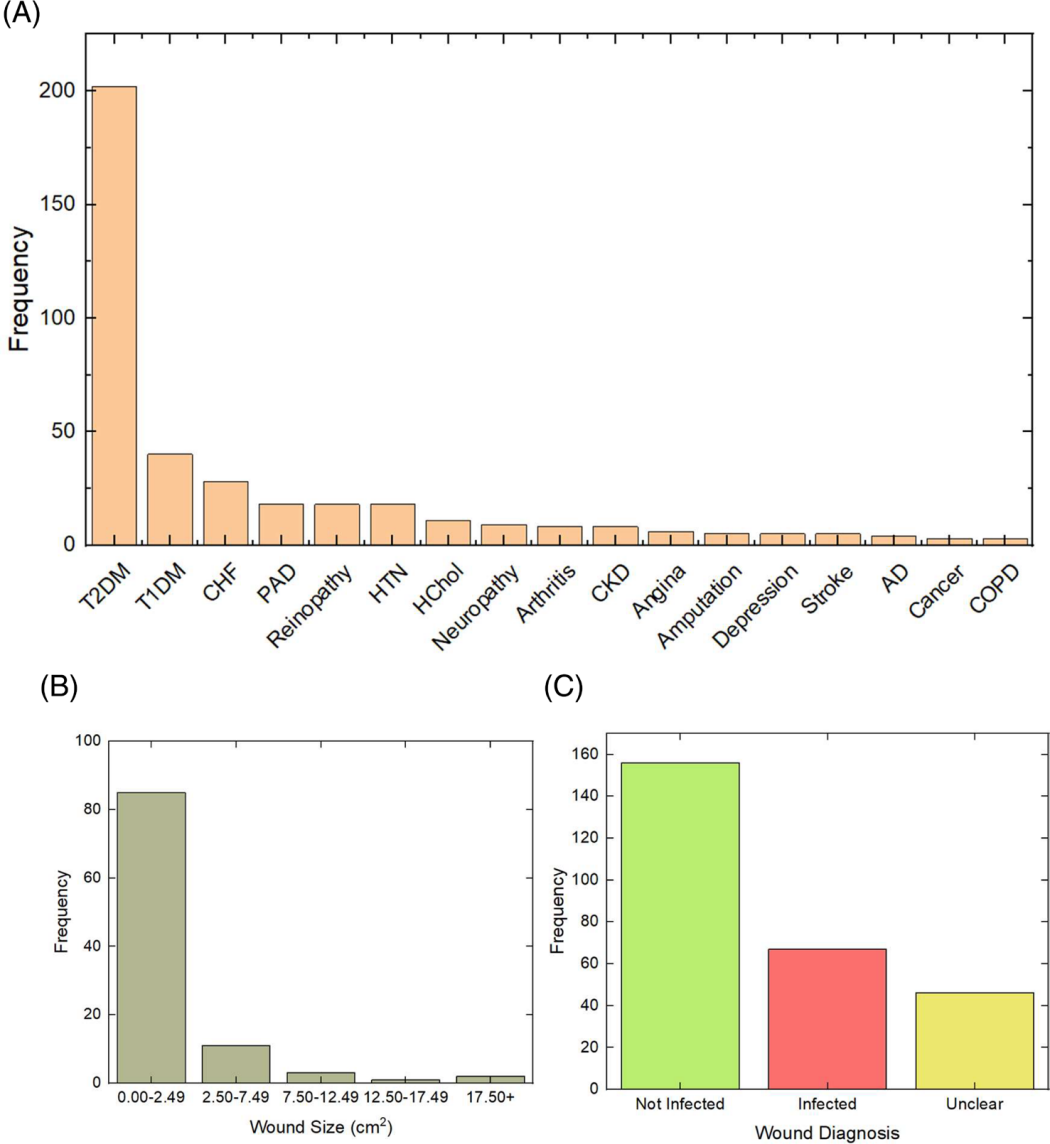
(a) Patient underlying conditions (b) size of wounds in the dataset and (c) Clinical infection diagnosis of the wounds.

Of the 267 patients, 60 were female, with an overall mean age of 68.7 years. Female patients made up a higher proportion of patients in the older age groups. 91% (*n* = 242) of the patients had diabetes. Other common underlying conditions included CHF, PAD, and HTN. A detailed breakdown of common underlying conditions in the data is provided in Figure 3a. Wound duration was mean 23.5 weeks and median 12.0 weeks. The mean was skewed upwards by 14 wounds with 100+ weeks duration. Mean wound size was 3.0 cm^2^, and median 1.0 cm^2^ [see Figure 3b].

Figure 3 (c) shows the clinical infection diagnosis for each wound. Crucially, in 17% (*n* = 46) of wounds, the clinician was unclear as to the infection status. The wound healing status was 28% (*n* = 60) healing, 33% (*n* = 70) indeterminate, and 40% (*n* = 85) not healing. This data was not reported in each case. Just under 75% of the ‘not healing’ wounds also reported infections or ‘unclear’ infection status.

**FIGURE 4:**
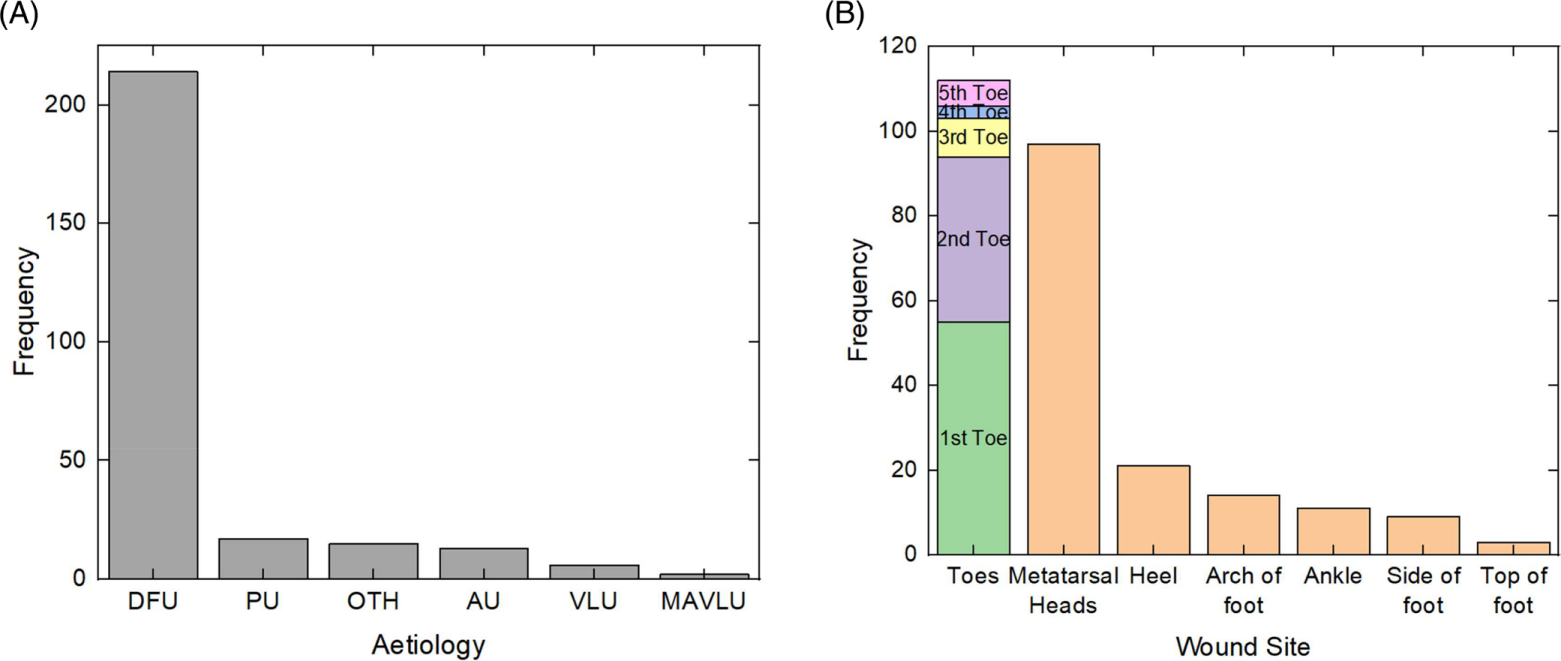
(a) Wound aetiology and (b) location of wounds.

Figure 4a breaks down the dataset by wound aetiology. DFUs accounted for 80% of all wounds. The breakdown of wound location is shown in Figure 4b. Most wounds were occurring on the toes.

### 4.2 Centre Versus Mean Wound Temperature Relationship

**FIGURE 5:**
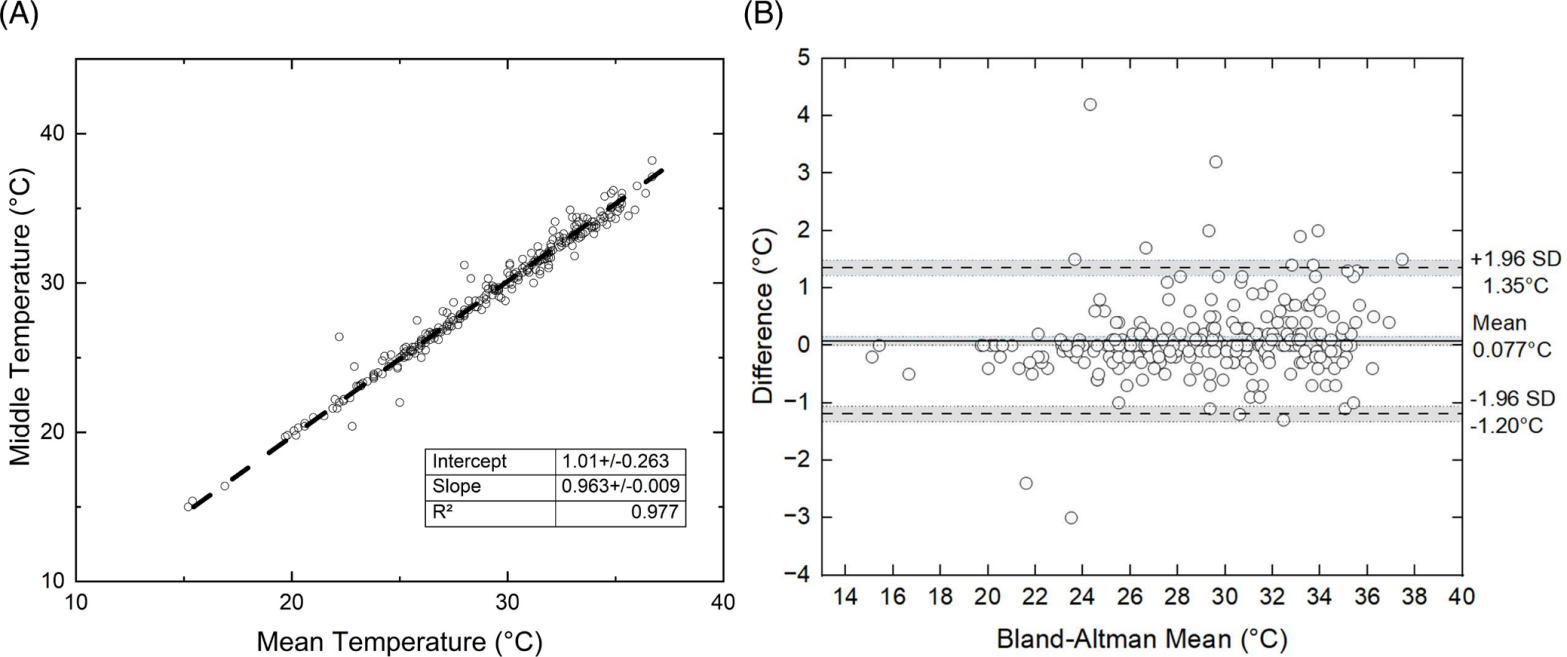
(a) Plots the *R* ^2^ analysis of the middle versus mean temperature data. (b) Shows a Bland–Altman plot of the middle versus mean temperature data.

Objective two of the study was to establish to what level the temperature in the centre of the wound correlates with the average temperature across the whole wound. The plot of middle temperature versus mean temperature in Figure 5a shows an *R* ^2^ value of 0.977.

Bland–Altman analysis of the mean temperature versus middle temperature is shown in Figure 5b. The dashed lines indicate the ±1.96 standard deviation from the mean; these lines define the limits of agreement. The shaded regions indicate the 95% CI. The mean difference value is 0.077°C, with an upper and lower 95% CI of 0.155°C and −0.001°C. The line of equality (0°C) lies within the CI, indicating that there is no significant systematic measurement difference between the middle wound temperature and the mean wound temperature value. The limits of agreement indicate that 95% of the measurements taken by the two techniques will have a difference between −1.20°C (95% CI −1.06°C to −1.33°C) and 1.35°C (95% CI 1.22°C to 1.49°C).

### 4.3 Temperature Difference as an Infection Indicator

**FIGURE 6:**
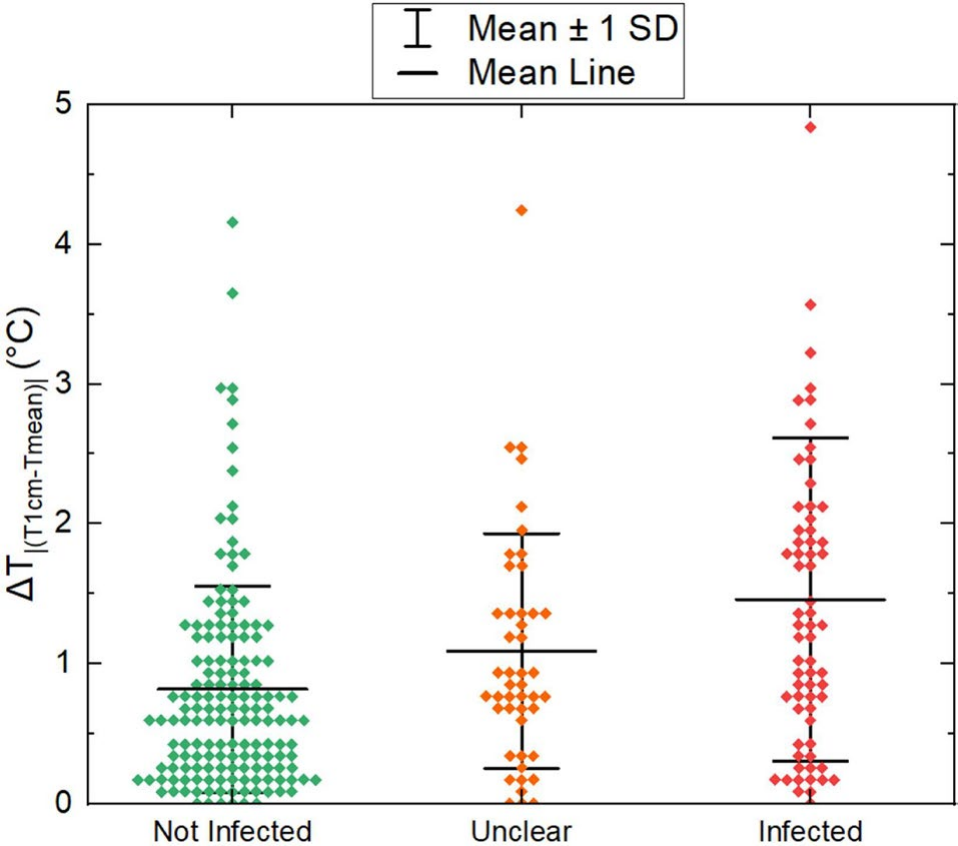
Whisker plot of the absolute difference in temperature (ΔT_|(T1cm‐Tmean)|_) between the average wound temperature and a point temperature measured 1 cm away from the edge of the wound. The whiskers mark ±1 SD. The centre line is the mean of the measurements.

The absolute value of the difference between mean wound temperature and periwound temperature (ΔT_|(T1cm‐Tmean)|_) is plotted in Figure 6 with the measurements categorised by infection status, with colour indicating infection status. The Pearson coefficient between infection status and ΔT_|(T1cm‐Tmean)|_ is 0.32.

### 4.4 Case Examples

**FIGURE 7:**
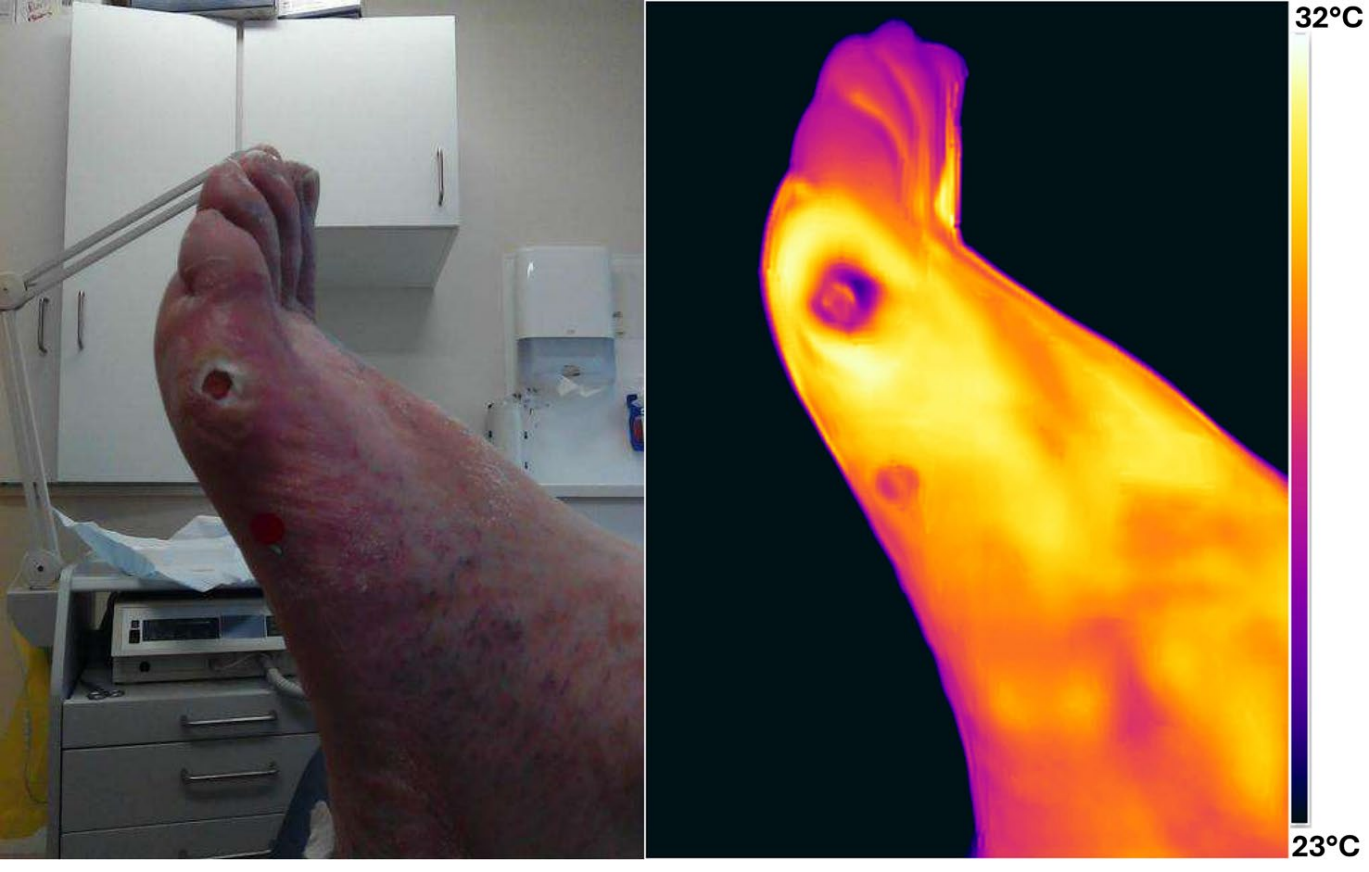
Visual and thermal image of ‘infected’ status wound.

Figure 7 shows visual and thermal images of a 67‐year‐old male with an ulcer on the lateral aspect of the fifth metatarsal head. The wound has been present for 16 weeks without any size reduction over the past 4 weeks. The patient has diabetes and venous insufficiency. The wound was debrided before capturing the image and was diagnosed as infected during the clinic visit.

The thermal image reveals a temperature difference of 3°C–4°C between the wound and the surrounding skin, which is indicative of an infection. Although some dead skin is visible around the wound, potentially causing artificially low temperature readings, the overall pattern shows a clearly elevated temperature in the periwound compared with the wound.

**FIGURE 8:**
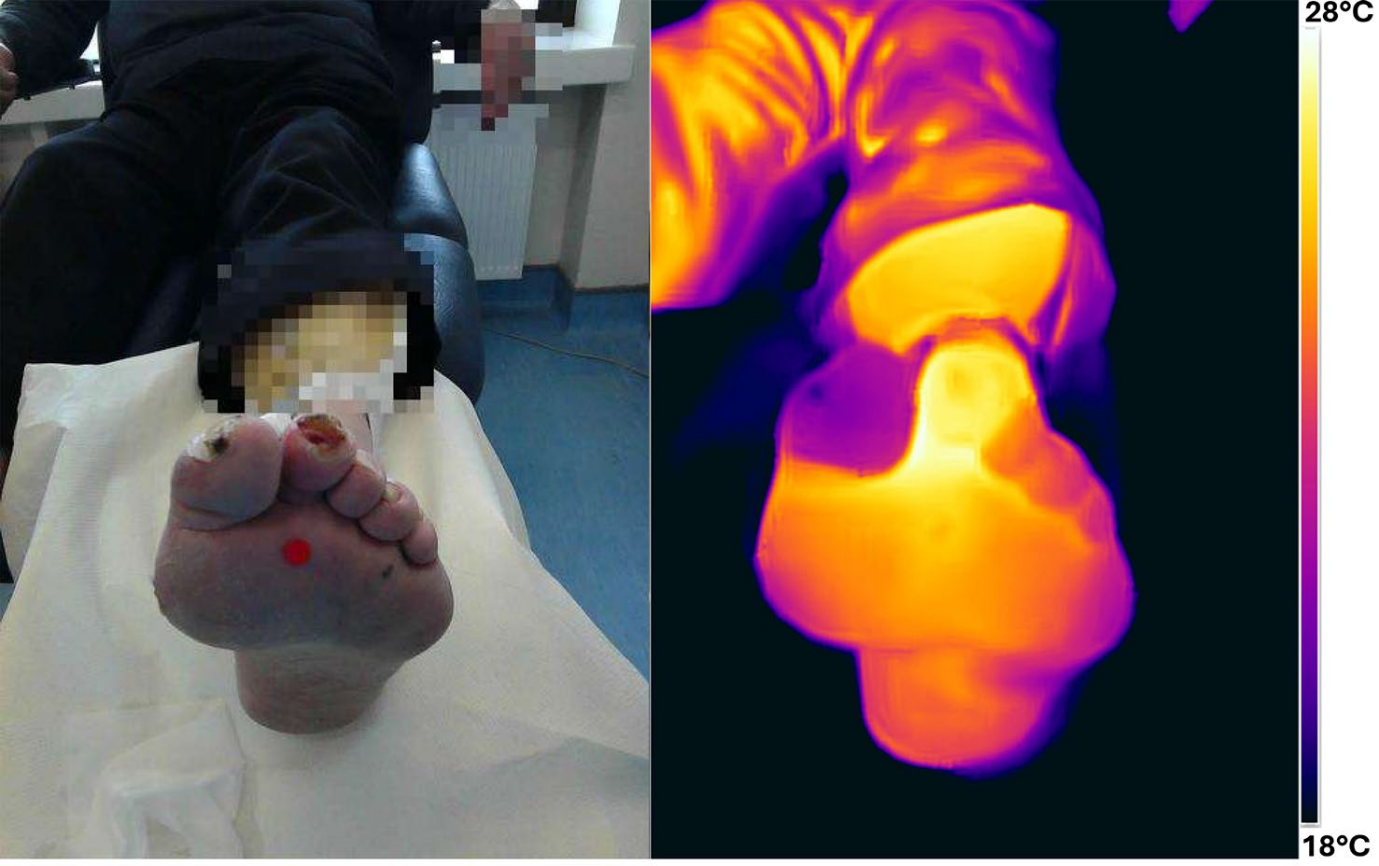
Visual and thermal image of ‘unclear’ status wound. Localised pixelation applied to visual image for patient privacy.

Figure 8 presents a visual and thermal image of an 84‐year‐old male with an ulcer on the second toe. The patient is diabetic and is suffering from osteomyelitis and PAD. The wound has been present for 7 weeks and has not reduced in size in the previous 4 weeks. The wound was debrided prior to taking the image. The infection status of the wound is 'unclear'. A 4°C–5°C temperature difference between the second toe and the other toes on the foot can be seen in the thermal image. This temperature difference is potentially indicative of an infected wound.

**FIGURE 9:**
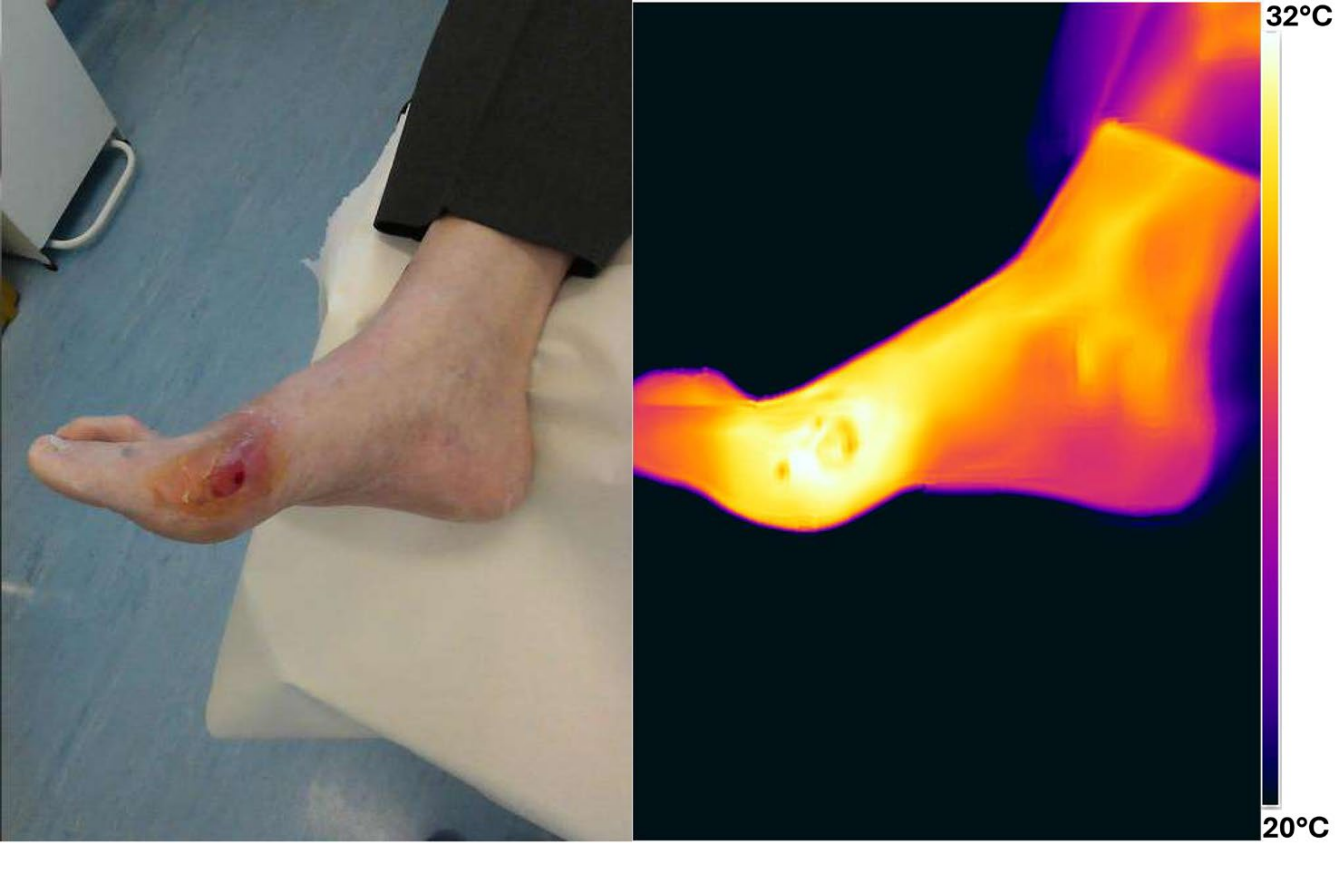
Visual and thermal image of ‘unclear’ status wound.

Figure 9 shows visual and thermal images of a 67‐year‐old male with an ulcer on the medial arch of the right foot. The patient has diabetes. The wound has been present for 8 weeks and has decreased in size by 40% over the past 4 weeks. The infection status of the wound is currently classified as'unclear'

The thermal image reveals a temperature difference of 2°C–3°C between the wound and the surrounding skin, which could be a sign of an infection.

## 5 Discussion

Clinical assessment of the wound bed relies on recording what the clinician can observe visually. Wound appearance can be documented and related to wound assessment charts such as Bates‐Jensen Wound Assessment Chart (BJWAT) and Wagner Grading Scale [18]. These recordings are mostly subjective and dependent on the clinician's expertise. This is a problem particularly when a patient is being referred from one care setting to another. Apart from measuring wound size using simplistic paper rulers or transparent grid dressings, no other objective methods of assessment are available and suited to routine clinics where most wound care practices are conducted. The assessment is unable to precisely identify the wound's stage of healing [19]. Such a determination would be clinically significant as chronic wounds become 'stuck' in the inflammation stage of healing. Determining if it has progressed beyond this is key for guiding treatment.

### 5.1 Objective 1

We have now developed a unique database that holds thermal images synchronised with photographs and correlated with clinician assessment for 267 wounds. There is potential to supplement this database further so that more robust statistical analysis can be used.

### 5.2 Objective 2

Objective 2 was to determine the relationship between the temperature at the centre of the wound and the average temperature of the entire wound. This relationship is visualised in Figure 6. The *R* ^2^ value from the plot in Figure 5a indicates that 97.7% of the variability in the middle temperature can be explained by the mean temperature. This shows a high level of correlation between the two sets of measurements.

The Bland–Altman Figure 5b shows that despite the high level of correlation between the two measurements, there is a considerable difference between the upper and lower limits of agreement in this case (2.55°C), although this difference can be mitigated somewhat by considering the confidence intervals. There does not appear to be any systematic differences between the measurements, or heteroscedasticity in the dataset. This discrepancy could be due to random errors in the measurements (outliers) which increase the limits of agreement. The temperature readings span a relatively large range (14°C–38°C), meaning discrepancies in measurements at the higher end of the temperature scale can disproportionately affect the limits of agreement. This dataset encompasses a variety of wound aetiologies, sizes, and patient comorbidities, all of which may influence the relationship between wound centre and mean temperatures.

### 5.3 Objective 3

Objective 3 examined the relationship between the mean wound temperature and the periwound temperature. As ΔT_|(T1cm‐Tmean)|_ increases, the wound is more likely to be infected, with a fair correlation (Pearson coefficient is 0.32). The correlation suggests that thermal imaging may assist in diagnosing infection in wounds in addition to the techniques already used.

We are not at the point to recommend routine use of thermal imaging to support clinical assessment of the wound, but our results indicate that with further research and development of a larger dataset, we may be close to such a recommendation. What is critical to understand is the meaning of the measurement together with how and where to take a measurement. Our results would indicate that taking the temperature at the centre of the wound is representative of the mean of the wound bed. This is important as a high level of inter‐rater reliability in identifying the centre of the wound has been shown, and such an approach is easy to teach and to understand [5]. Secondly, in our current study, we have shown a fair correlation between wound temperature and wound status, and this will help us to further understand the meaning of the measurement and if thresholds exist within which a wound can be said to have a ‘normal’ temperature.

An important finding of our study was that in almost 20% of observations clinicians were unclear as to the infection status of the wound. This has implications for decision making and arguably in the prescribing of antibiotics when in doubt. Our aim is to support the assessment process for these 20% of cases and not to waste resources and time in recording temperature when it is obvious to the clinician the wound status.

The ultimate test of any diagnostic tool is a randomised controlled trial that would incorporate knowledge of wound temperature into the wound assessment process versus no temperature and evaluate this in terms of healing outcomes or other appropriate outcomes. We would welcome such a study.

While we did not seek to explicitly replicate the Glamorgan Protocol in our methods for data capture, our process aligns well with it. As thermal images were to be taken as part of routine clinic visits, patients were not advised to avoid smoking or exercise prior to attending, although such advice is part of routine care. All patients were seated in a waiting area prior to the clinical procedures. The camera was allowed time to warm up prior to taking images and was calibrated with a reference point. Methods of image capture were standardised, and we have reported on methods of data capture and analysis in the methods section. Clinicians taking images were trained in the use of the device. We welcome the testing and development of such protocols, as this will ensure that any device for future use in clinical assessment should be of the highest possible standards to enhance validity and reliability.

## 6 Conclusion

This work has established two correlations, which can be used to assist clinical diagnosis of chronic wounds. There is a strong correlation between the temperature at the centre of the wound and the average temperature of the whole wound, and an absence of systematic bias. There is some measurement uncertainty represented in the broad limits of agreement in Figure 5b. Our conclusion is that the middle temperature is representative of the mean, making it suitable as a supplementary tool for wound diagnostics.

The second correlation is that the difference in temperature between the centre of the wound and the healthy skin around the wound shows reasonable correlation with the infection status of the wound. In the case where a clinician is struggling to make an infected or not infected diagnosis of a wound, a low‐cost thermal imager could be used as an additional diagnostic tool. Thermal imaging is a low‐cost, non‐contact, instant, and easy‐to‐use tool for clinicians when examining chronic wounds. It should be used as a supplement to a clinician's expertise, enhancing their ability to assess wound conditions and make informed decisions.

Future work further analysing the thermal image dataset using image analysis tools and machine learning techniques to enhance infection detection processes is envisaged.

## Conflicts of Interest

The authors declare no conflicts of interest.

## Data Availability

The data that support the findings of this study are openly available in figshare at https://doi.org/10.6084/m9.figshare.29660651.v1.
